# Carrageenan/Chitin Nanowhiskers Cryogels for Vaginal Delivery of Metronidazole

**DOI:** 10.3390/polym15102362

**Published:** 2023-05-18

**Authors:** Natallia V. Dubashynskaya, Valentina A. Petrova, Andrey V. Sgibnev, Vladimir Y. Elokhovskiy, Yuliya I. Cherkasova, Yury A. Skorik

**Affiliations:** 1 Institute of Macromolecular Compounds, Russian Academy of Sciences, Bolshoy pr. V.O. 31, St. Petersburg 199004, Russiavalentina_petrova_49@mail.ru (V.A.P.);; 2 Institute for Cellular and Intracellular Symbiosis, Ural Branch of the Russian Academy of Sciences, Pionerskaya st. 11, Orenburg 460000, Russiayulya.cherkasova.2018@mail.ru (Y.I.C.)

**Keywords:** carrageenan, chitin nanowhiskers, cryogels, metronidazole, vaginal delivery, trichomoniasis

## Abstract

The development of polymeric carriers based on partially deacetylated chitin nanowhiskers (CNWs) and anionic sulfated polysaccharides is an attractive strategy for improved vaginal delivery with modified drug release profiles. This study focuses on the development of metronidazole (MET)-containing cryogels based on carrageenan (CRG) and CNWs. The desired cryogels were obtained by electrostatic interactions between the amino groups of CNWs and the sulfate groups of CRG and by the formation of additional hydrogen bonds, as well as by entanglement of carrageenan macrochains. It was shown that the introduction of 5% CNWs significantly increased the strength of the initial hydrogel and ensured the formation of a homogeneous cryogel structure, resulting in sustained MET release within 24 h. At the same time, when the CNW content was increased to 10%, the system collapsed with the formation of discrete cryogels, demonstrating MET release within 12 h. The mechanism of prolonged drug release was mediated by polymer swelling and chain relaxation in the polymer matrix and correlated well with the Korsmeyer–Peppas and Peppas–Sahlin models. In vitro tests showed that the developed cryogels had a prolonged (24 h) antiprotozoal effect against *Trichomonas*, including MET-resistant strains. Thus, the new cryogels with MET may be promising dosage forms for the treatment of vaginal infections.

## 1. Introduction

One of the most common strategies to improve the efficacy of known drugs is their modification by incorporation into various polymeric systems based on natural polysaccharides [1,2]. For example, the use of polymeric carriers improves vaginal delivery due to prolonged and controlled drug release, increased local bioavailability of antimicrobial agents, prolonged drug residence time at target sites due to the bioadhesive properties of polysaccharides [3,4], reduced toxicity to normal microflora, and synergy/potentiation of action due to the antimicrobial effects of polymers such as chitin, chitosan, and its derivatives [5,6,7,8]. Hydrogels are widely used as polymeric drug carriers in gynecology. In general, hydrogels are polymeric networks consisting of macromolecular chains held together by physical interactions (e.g., hydrogen bonding and chain entanglement), as well as by chemical crosslinking. However, pure polysaccharide hydrogels do not have the necessary mechanical properties, high drug loading, and programmed drug release. These disadvantages can be overcome by modifying the hydrogel structure with crosslinking or doping agents [9,10]. In this context, hydrogels based on hydrophilic natural polysaccharides, including those modified by chitin nanofibers (chitin nanowhiskers, CNWs), are an attractive composite dosage form for vaginal applications [11,12]. However, hydrogels as a liquid dosage form are less chemically and microbiologically stable than their anhydrous analogs, such as cryogels obtained by lyophilic drying. Polysaccharide-based cryogels have a spongy supermacroporous structure after lyophilization, which swells in contact with biological fluids to form a hydrogel layer [13]. Polysaccharide cryogels are characterized by modified release, mucoadhesion, and high stability; they are perfect candidates for the development of vaginal dosage forms with improved biopharmaceutical properties [14].

CNWs are the product of partial deacetylation of the nitrogen-containing biopolymer α-chitin, which consists of N-acetylglucosamine residues linked by β-(1→4)-glycosidic linkages. As a result of partial deacetylation, D-glucosamine residues (not more than 50%) appear in the macromolecule. These modified polysaccharide molecules contain active cationic amino groups (surface cationization), and at the same time, they are insoluble in both water and acidic solutions. Thus, CNWs retain the beneficial properties of chitin and chitosan, demonstrating excellent biocompatibility, biodegradability, low immunogenicity, and antibacterial activity [15,16]. In addition, CNWs are an active filler that not only changes the mechanical properties, structure, morphology, and biodegradability of the material, but also modifies the drug release [11,17,18]. The biphasic nature of this type of composite hydrogel determines its advantages when used as a drug carrier for active pharmaceutical ingredients, allowing the programming and regulation of drug release [17].

For example, Lin et al. [18] developed sodium-alginate-based microspheres formed by ionic crosslinking and reinforced with CNWs. The resulting microspheres exhibited controlled swelling patterns, high encapsulation efficiency, and promising prolonged drug release profiles. In addition, CNWs improved the strength and integrity of the microspheres compared to CNW-free particles. Our research group has developed polysaccharide systems for the delivery of antimicrobial agents in the form of sodium alginate/CNW hydrogels with tetracycline, as well as in the form of metronidazole (MET)-containing microgels based on polyelectrolyte complexes of sodium alginate and CNWs [11,17]. Composite systems based on sodium alginate and CNWs were formed due to complex interactions: (i) electrostatic interaction between the negatively charged carboxylate groups of sodium alginate and the positively charged amine groups of partially deacetylated chitin, (ii) hydrogen bonding, and (iii) mechanical entanglement of sodium alginate macrochains. It has been shown that by varying the CNW content, we can control the viscosity of the resulting systems and the rate of tetracycline release [17]. In addition, sodium alginate/CNW PEC-based systems also exhibited prolonged pH-sensitive release of MET (37 and 67% of the drug were released within 24 h at pH 7.4 and pH 4.5, respectively) [11].

Carrageenan (CRG)-based hydrogels are of interest as vaginal delivery systems for the treatment of various sexually transmitted infections because the spectrum of activity of sulfated polysaccharides extends to various enveloped viruses, including herpes simplex virus, cytomegalovirus, and human immunodeficiency virus [19]. CRGs consist of residues of D-galactose and its derivatives linked by regularly alternating β(1→4) and α(1→3) glycosidic linkages. The structural diversity of CRGs (κ-, ι-, λ-CRGs, etc.) is due to the presence of a β-(1-4)-linked monosaccharide residue in the form of 3,6-anhydrogalactose, as well as the number and location of sulfate groups in the molecule [20,21,22]. The ability to form gels in aqueous solutions is an important physicochemical property of CRGs. Gel formation is due to intermolecular interactions in which each macromolecule cooperatively associates with several others. The result is a single three-dimensional network of dissolved polymer molecules and a large amount of solvent [23]. Upon freeze drying, such gels form porous cryogel structures, which are suitable for practical use as drug delivery systems due to their stability [13,24]. In addition, upon contact with the mucosa, cryogels swell and provide mucoadhesion and sustained controlled drug release [25,26]. The gelling properties of CRGs depend on their chemical structure; they increase with decreasing degree of sulfation and increasing content of 3,6-anhydrogalactose [21]. CRGs also have excellent mucoadhesive properties, which are useful in dosage forms intended for mucosal application [4,27]. The presence of oppositely charged functional groups in CRGs and CNWs allows the formation of polyelectrolyte complexes and regulates the morphology, structure of hydrogels, and their biopharmaceutical properties [11,17,28].

Topical application of drugs provides effective therapeutic concentrations directly at the target site and thus increased local bioavailability, which allows reducing drug dose and side effects [29]. Vaginal drug delivery is one of the most important routes of drug administration for various gynecological pathologies, both inflammatory diseases and sexually transmitted infections (e.g., trichomoniasis, ureaplasmosis, candidiasis, etc.) [30]. Trichomoniasis, caused by an extracellular eukaryotic parasite, the protozoan *Trichomonas vaginalis*, is the most common non-viral sexually transmitted infection [31]. In addition, *T. vaginalis* infection may contribute to human immunodeficiency virus transmission [32], increase the risk of cervical cancer [30], and be associated with adverse pregnancy outcomes and infertility [33]. The drug of choice for the treatment of trichomoniasis is usually MET, an imidazole derivative with antiprotozoal and antimicrobial activity [34]. A more serious challenge is the increasing resistance of *Trichomonas* to antibacterial agents, including MET [35].

Effective treatment of vaginal infections is complicated by several anatomical and physiological factors. The sinuosity of the vaginal canal and the many folds in the vaginal walls make it difficult to evenly distribute traditional vaginal dosage forms, such as vaginal gels or showers [36]. In addition, cervicovaginal mucus can complicate the penetration, distribution, and residence time of therapeutic agents at target sites [37]. Therefore, traditional vaginal dosage forms have several limitations to their high efficacy (e.g., uneven distribution, short vaginal residence time, discomfort, and low patient compliance), necessitating the development of new drug delivery systems with improved functionality [38].

The aim of our study was to develop a CNW-modified CRG-based cryogel for sustained and controlled release of MET. The modified delivery will increase the efficiency of administering MET, reduce its dosage, and prevent microbial resistance from developing. These cryogels have promising potential for the treatment of vaginal infections. To the best of our knowledge, there are no data on the development of CRG/CNW-based cryogel structures for vaginal delivery of MET.

## 2. Materials and Methods

### 2.1. Materials

In this study, k/β-CRG (Sigma-Aldrich, St. Louis, MI, USA) with a sulfur content of 4.8% was used. The sulfur content was determined by inductively coupled plasma mass spectrometry (ICP-MS) using an Agilent ICP-MS 8800 (Agilent Technologies, Santa Clara, CA, USA). The viscosity of the 1% k/β-CRG solution was 969 cP. Dynamic viscosity was measured using a Fungilab viscometer (Fungilab, S.A., Barcelona, Spain).

CNWs were obtained by partial deacetylation of α-chitin with a particle size of 0.1–0.2 mm, as previously reported [18,39]. The degree of deacetylation (DDA 0.40) was determined by conductometric titration and by elemental analysis. The size of the CNWs (thickness 6–15 nm, length 100–300 nm) was estimated by scanning electron microscopy [39].

The MET, sodium chloride, potassium hydroxide, calcium hydroxide, bovine serum albumin, glycerol, urea, and glucose were from Sigma-Aldrich (St. Louis, MI, USA); the 1 M hydrochloric acid solution, lactic acid, and acetic acid were from Acros Organics (Waltham, MA, USA). All of the chemicals were analytical grade.

Vaginal fluid simulant (VFS) was prepared from sodium chloride (3.51 g/L), potassium hydroxide (1.40 g/L), calcium hydroxide (0.222 g/L), bovine serum albumin (0.018 g/L), lactic acid (2 g/L), acetic acid (1 g/L), glycerol (0.16 g/L), urea (0.4 g/L), glucose (5 g/L). The pH of the resulting mixture was adjusted to pH 4.5 with 0.1 M HCl [40].

### 2.2. Preparation of CRG-CNW Hydrogels (CRG-CNW-HG)

To obtain hydrogels based on CRG and CNWs (CRG-CNW-HG), CRG was dissolved in the calculated amount of water, and then these concentrated CRG solutions were mixed with a 0.5% aqueous dispersion of CNWs (Table 1). The resulting CRG-CNW dispersion was homogenized for 1 h under mechanical stirring, and then 0.2 mL of 2% acetic acid solution was added under strong stirring. Hydrogels with a CRG mass of 0.8 g and CNW content of 3, 5, and 10% of the CRG mass (CRG-CNW-HG-3, CRG-CNW-HG-5, and CRG-CNW-HG-10, respectively) were obtained. CNW-free hydrogel (CRG-HG) was obtained by the same technique and used as a control (Table 1). The pH values of the obtained hydrogels were 5.3 ± 0.2. The hydrogels were incubated at room temperature for 1 day and then stored in a refrigerator at 4 °C for 2 days.

### 2.3. Preparation of CRG-CNW Cryogels (CRG-CNW-CG)

To convert the obtained systems (CRG-HG, CRG-CNW-HG-3, CRG-CNW-HG-5, and CRG-CNW-HG-10) into cryogel form, the formed hydrogels were freeze dried using a 10 N freeze dryer (Fanbolun Ltd., Guangzhou, China). As a result, cryogels with different CNW contents were obtained: CRG-CG, CRG-CNW-CG-3, CRG-CNW-CG-5, and CRG-CNW-CG-10.

### 2.4. Isolation of CRG-CNW Microparticles (CRG-CNW-MP) from CRG-CNW-HGs

To isolate CRG-CNW microparticles (CRG-CNW-MP), CRG-CNW-HGs were diluted with water to break the physically bound gel [11]. The microparticles were separated from the CRG solution by centrifugation using an MPW-308R centrifuge (MPW Med. Instruments, Warszawa, Poland) at 4500 rpm, washed with water, and freeze dried. To measure hydrodynamic radii and ζ-potentials, the microparticles were redispersed in water at a concentration of 1 mg/mL and stirred for 24 h. The resulting suspensions were diluted to a concentration of 0.1 mg/mL, and large aggregates were separated by centrifugation (2 min, 2000 rpm).

### 2.5. Preparation of MET-Loaded CRG-CNW-CGs (MET-CRG-CNW-CG)

MET-containing hydrogels were obtained by introducing MET at the stage of preparing CRG solutions (Table 1). First, MET (0.267 g) was dissolved in water and then 0.8 g of CRG was added. CNWs and 2% acetic acid solution were added as described in Section 2.2. Second, the obtained systems were freeze dried, resulting in MET-containing cryogels: MET-CRG-CG, MET-CRG-CNW-CG-5, MET-CRG-CNW-CG-10. The MET content (µg/mg) was calculated according to the following equation:

(1) $$\mathrm{MET}\;\mathrm{content}\;(\mu g/\mathrm{mg})=\frac{m\left(\mathrm{MET}\right)\times 1000}{m\left(\mathrm{CRG}\right)+m\left(\mathrm{CNW}\right)+m\left(\mathrm{MET}\right)}.\;$$

### 2.6. General Methods

#### 2.6.1. Elemental Analysis

Elemental analysis was performed using a Vario EL CHN analyzer (Elementar, Hanau, Germany).

The molar ratio between the monomeric units of CRG and CNWs was estimated using elemental analysis data and the following Equation (2):

(2) $$\mathrm{CRG}:\mathrm{CNW}=\frac{1}{x}\left[{\left(\frac{C\%}{N\%}\right)}_{\mathrm{CRG}-\mathrm{CNW}-\mathrm{MP}}-{\left(\frac{C\%}{N\%}\right)}_{\mathrm{CNW}}\right]\frac{MW_{N}}{MW_{C}}\;,$$

where *x* is the number of C atoms in the monomeric units of CRG (*x* = 6); *C*% and *N*% are the mass fractions of the corresponding elements (CNW: *C*% = 43.0%, *N*% = 7.0%; CRG-CNW-MP: *C*% = 38.2%, *N*% = 4.9%); *MW* is the molecular weight of the corresponding element.

#### 2.6.2. Rheological Properties

The rheological properties of the hydrogels were studied using a Physica MCR301 rheometer (Anton Paar GmbH, Graz, Austria) in a CC17-SN11329 (ISO 3219) cylindrical measuring device in dynamic (sinusoidal) and shear modes with decreasing (Down) and increasing (Top) strain rate (circular frequency) at 20 °C.

To describe the rheological behavior of the samples, we used the Cross equation with a conditional yield stress characterizing the strength of the supramolecular structure, since the shear test in the Down SR mode assumes the most disrupted gel structure, where the system behaves as a structured liquid:

(3) $$\tau \left(\dot{\gamma }\right)=\tau_{0}+\eta_{\infty }\dot{\gamma }+\frac{\left(\eta_{0}-\eta_{\infty }\right)\dot{\gamma }}{1\;+\;{\left(\theta \dot{\gamma }\right)}^{p}}\;\;\eta \left(\dot{\gamma }\right)=\frac{\tau_{0}}{\dot{\gamma }}+\eta_{\infty }+\left(\eta_{0}-\eta_{\infty }\right){\;\left[1\;+\;{\left(\theta \dot{\gamma }\right)}^{p}\right]}^{-1}$$

where $\tau \left(\dot{\gamma }\right)$ is the shear stress (Pa); *τ_0_* is the conditional yield stress; $\eta \left(\dot{\gamma }\right),$ $\eta_{0}$*,* $\eta_{\infty }$ are the effective viscosity, maximum, and minimum Newtonian viscosity, respectively (Pa·s); θ is the relaxation time (s); *p* is the power index (for many polymers, this is equal to 2/3).

#### 2.6.3. X-ray Diffraction

X-ray diffraction analysis was performed with a DRON-3M instrument (Burevestnik, St. Petersburg, Russia) using Ni-filtered Cu Kα radiation (λ = 1.5418 Å).

#### 2.6.4. Scanning Electron Microscopy

The morphology of the cryogels was studied by scanning electron microscopy (SEM), which was performed on a Tescan Mira 3 scanning electron microscope (Tescan, Brno, Czech Republic). Freeze-dried samples were placed on double-sided carbon tape for SEM studies. Images were acquired in the secondary electron mode at an accelerating voltage of 4 kV and an operating current of 40 pA; the distance between the sample and the detector was 7–8 mm.

#### 2.6.5. Light Scattering

The hydrodynamic radii and ζ-potential of the CNWs and CRG-CNW-MPs were measured using a Photocor Compact-Z instrument (Photocor Ltd., Moscow, Russia) with a 659.7 nm He-Ne laser at 25 mV power and a detection angle of 90°.

#### 2.6.6. Specific Surface Area

The specific surface area was determined by measuring the Kr adsorption isotherm at 77 K with a QuadraSorb instrument (Quantachrome Instruments, Boynton Beach, FL, USA) using the Brunauer, Emmett, and Teller (BET) method. All samples were measured after degassing at 70 °C.

### 2.7. MET Release Kinetics

To study drug release, VFS (pH 4.2 and temperature 37 ± 1 °C) was used as the release medium to simulate the physiological characteristics of the vaginal environment (acidic pH due to the presence of lactic acid and body temperature) [37].

10 mg of the sample was placed in a plastic tube with a dialysis membrane attached to the end. The tube was immersed in a glass containing 30 mL of VFS (37 ± 1 °C) under constant stirring. At intervals (0.5, 1, 2, 3, 5, 7, 10, and 24 h), 2 mL of the release medium was sampled for spectrophotometric determination of MET concentration at 320 nm with a calibration curve using a UV-1700 PharmaSpec spectrophotometer (Shimadzu, Kyoto, Japan). The sampled volume was replaced with 2 mL of fresh VFS. Each sample was tested in triplicate (*n* = 3).

### 2.8. Pharmacological Activity of MET-CRG-CNW-CG

Thirty strains of *T. vaginalis*, twenty-nine clinical isolates, and one MET-resistant strain from the American Type Culture Collection (ATCC 50143), were used to study the pharmacological activity of cryogels. *T. vaginalis* strains were cultured in tubes containing 5 mL of Diamond’s trypticase–yeast–maltose medium (TYM, Scharlab, Barcelona, Spain) supplemented with 10% heat-inactivated fetal bovine serum (FBS, Capricorn Scientific GmbH, Ebsdorfergrund, Germany) [41]. An antimicrobial cocktail (final concentrations) of amikacin (4 µg/mL), amphotericin B (5 µg/mL), ampicillin (1 mg/mL), chloramphenicol (1 µg/mL), ciprofloxacin (2 µg/mL), and vancomycin (5 µg/mL) (Sigma-Aldrich, St. Louis, MI, USA) was added to reduce contamination of the medium by microorganisms associated with *Trichomonas*. The pH of the TYM was adjusted to pH 4.2 with 0.1 M HCl.

Clinical strains were isolated from vaginal fluid samples collected from fertile women (18–45 years of age) attending the clinic of the Orenburg State Medical University (Russia) from August to December 2022. This phase of the study was conducted in accordance with Good Clinical Practice guidelines and approved by the Human Research Ethics Committee of the Orenburg State Medical University. All patients gave written informed consent to participate in the study.

Vaginal specimens were collected with a sterile swab and transported in a temperature-controlled container at 37 °C using an anaerobic swab transporter. Swabs were inoculated into prewarmed (37 °C) TYM within 2 h of collection. Cultures of clinical isolates were examined daily from day 2 to day 7 by wet slide microscopy. *T. vaginalis* trophozoites were identified by their size (10–20 µm), oval shape, and characteristic motility [42].

All clinical isolates were then passaged by transferring 500 μL of culture to 5 mL of fresh TYM at 48 h intervals until uncontaminated axenic cultures were obtained. The presence of bacteria or yeast was checked by inoculating all isolates (100 μL) in a liquid medium containing thioglycolate and on blood agar plates (37 °C, 5% CO_2_, for 3 days).

After obtaining axenic cultures, susceptibility analysis to MET was performed [43]. MET susceptibility testing was performed in 96-well flat-bottomed microtiter plates in 5% CO_2_ at 37 °C. Two-fold serial dilutions of MET were performed in TYM medium. The resulting concentrations ranged from 0.125 to 32 µg/mL. Cultures of *T. vaginalis* were standardized to an inoculum of 10^5^ *Trichomonas* using a hemocytometer. 90 μL of the MET dilution was added to each well, followed by 10 μL of the inoculum of each *T. vaginalis* isolate. TYM without MET was used as a control. Plates were incubated in 5% CO_2_ at 37 °C for 48 h. Motility and growth of *T. vaginalis* were assessed using an inverted microscope at ×400 magnification. The minimum inhibitory concentration (MIC) was defined as the lowest concentration (µg/mL) of MET that did not produce motile trophozoites after 48 h of incubation. All experiments were performed in triplicate for each strain of *T. vaginalis*.

To study the duration of the antiprotozoal effect of MET-CRG-CNW-CG, the residual antiprotozoal activity (RAA) of the cryogel was measured. The amount of MET that remained in the cryogel after its preincubation in TYM for a certain period of time was taken as RAA. To measure RAA, 1 mg of cryogel was placed in an ultrafiltration tube with a dialysis membrane insert (3 kDa MWCO) complete with a centrifuge tube containing 3 mL of TYM. The tubes were incubated at 37 °C with constant shaking. At intervals of 1, 2, 3, 4, 5, 7, 10, and 24 h, TYM was removed by centrifugation without breaking the gel (100 g, 2 min). The cryogel was then transferred to a tube containing 3 mL of fresh TYM and incubated at 37 °C for a time defined as the difference between the pre-incubation interval and 24 h, after which the cryogel was pelleted by centrifugation at 12,000× *g*, 10 min. The supernatant was additionally sterilized by filtration (0.22 μm), then a series of two-fold dilutions were prepared from it in TYM and used to determine RAA in the same manner as for MET. RAA was expressed in arbitrary units (a.u.) and was determined as the number of two-fold dilutions in the last of which there was still an antiprotozoal effect [44]. All experiments were performed in triplicate for each clinical isolate and in nine cases for *T. vaginalis* ATCC 50143.

Survival of *T. vaginalis* was estimated using the Kaplan–Meier method; survival curves were compared using the log-rank (Mantel–Cox) and Gehan–Breslow–Wilcoxon tests; median survival and the log-rank test for trend were also evaluated. All reported *p* values were two-tailed; a value of *p* < 0.05 was considered statistically significant.

## 3. Results and Discussion

### 3.1. Structure of Polysaccharides

**Figure 1:**
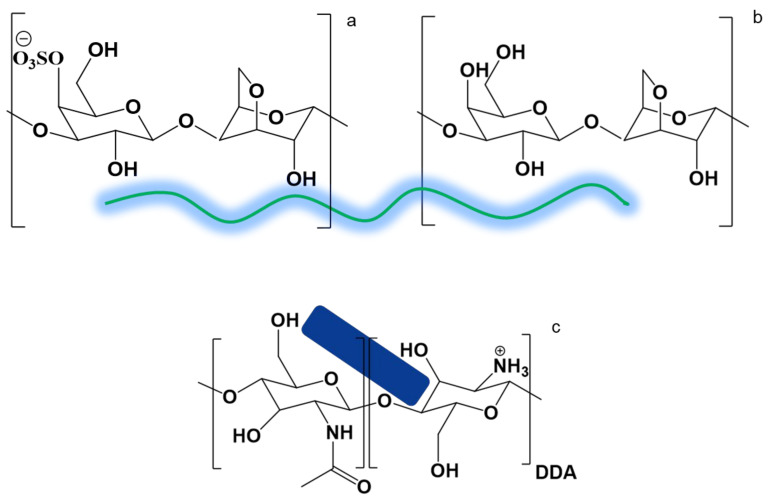
Chemical structure of polysaccharides: κ-CRG (**a**), β-CRG (**b**), CNW (**c**).

One of the most common types of gelling CRGs is κ-CRG, whose structural unit is a disaccharide consisting of alternating O3-substituted β-D-galactopyranosyl and O4-substituted 3,6-anhydro-α-D-galactopyranosyl residues, with most D-galactose units containing a sulfate half-ester group at O4 (one sulfate group per disaccharide unit) (Figure 1a) [45]. Natural CRGs are often hybrids, such as κ/β-CRG [21,46,47]. Unlike κ-CRG, β-CRG does not contain the O4-sulfate group in the galactose subunit (Figure 1b) [48].

CNWs obtained by partial deacetylation of α-chitin contain amino groups (pKa ~ 6.5) on their surface, which are protonated and become positively charged in an acidic environment (Figure 1c). Due to the high specific surface area and positive surface charge, the CNWs can be ionically bound with negatively charged sulfate groups of CRG to obtain various drug carriers with desired properties, structure, and morphology [11,17,18].

### 3.2. Preparation and Properties of CRG-CNW-CG and CRG-CNW-MP

The formation of a two-phase CRG-CNW composite hydrogel occurred both by the formation of a polyelectrolyte complex due to the electrostatic interaction of negatively charged sulfate groups of CRG with positively charged amine groups of CNW and the formation of additional hydrogen bonds, and by the formation of a physical gel due to the entanglement of macromolecular chains. When water is added to the hydrogel, the physical gel slowly disintegrates and most of the CRG passes into the solution containing the CRG-CNW microparticles (microgel). To study the composition, the microparticles were isolated from the microgels by centrifugation, and the molar ratio between the monomeric units of CRG and CNWs was estimated using elemental analysis data.

According to the elemental analysis data, the obtained polyelectrolyte complex CRG-CNW-MP has the ratio CRG:CNW = 0.33, i.e., almost all amino groups of CNWs with DDA of 0.40 are bound to CRG.

According to electrophoretic light scattering data, in contrast to positively charged CNWs (ζ-potential = 20 ± 2 mV), CRG-CNW-MPs had a negative ζ-potential of about −13.4 ± 0.3 mV due to the presence of CRG molecules.

Dynamic light scattering showed that CNWs had apparent hydrodynamic diameters (*D*_h_) of 50 ± 30 and 300 ± 10 nm, which was in good agreement with the SEM data [39]. CRG-CNW-MPs also had two sizes with *D*_h_ = 56 ± 12 and 374 ± 108 nm, indicating the preservation of the dominant CNW structure in the resulting systems, which is characteristic of various objects with CNWs [11].

**Figure 2:**
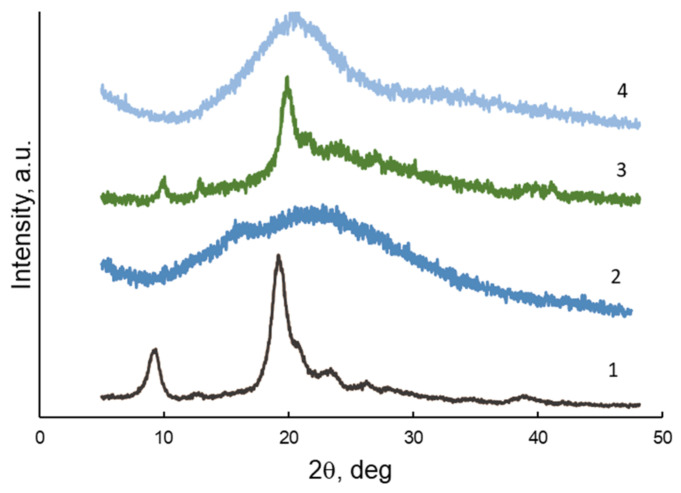
X-ray diffraction patterns of the samples: CNW (1), CRG (2), CRG-CNW-MP (3), CRG-CNW-CG (4).

X-ray diffraction analysis of microparticles and hydrogels (Figure 2) also showed that CRG-CNW-MPs (3) retained the structure of the original CNWs (1) with specific but less pronounced reflections in the region of 2θ = 20° and 10°. However, their structure is more amorphous due to the presence of amorphous CRG (2). The structure of CRG-CNW-HGs (4) had a diffraction pattern different from that of CRG (2), with a more pronounced signal in the region of 2θ = 20°, indicating the contribution of CNWs to the structural organization of the hydrogel.

**Figure 3:**
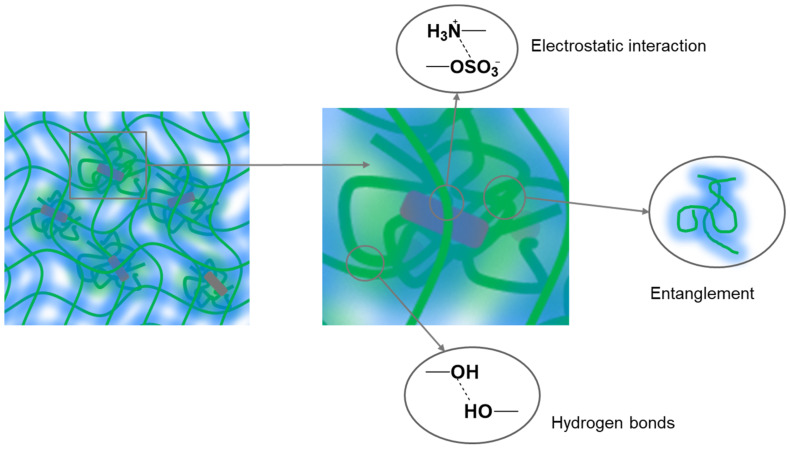
Schematic representation of hydrogel matrices based on CRG structured with CNWs.

Thus, when CRG interacts with CNWs in an aqueous solution (pH = 5.3), a complex biphasic hydrogel system is formed consisting of a three-dimensional network of CRG macromolecules with CNWs entrapped within (Figure 3).

### 3.3. Rheological Properties of CRG-HG and CRG-CNW-HGs

**Figure 4:**
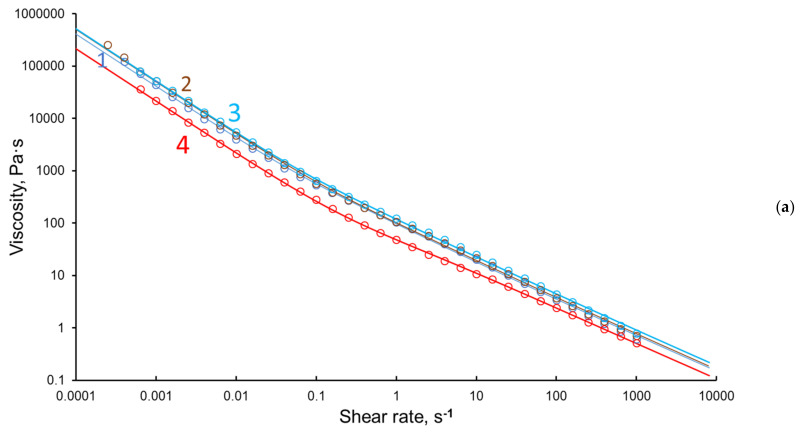

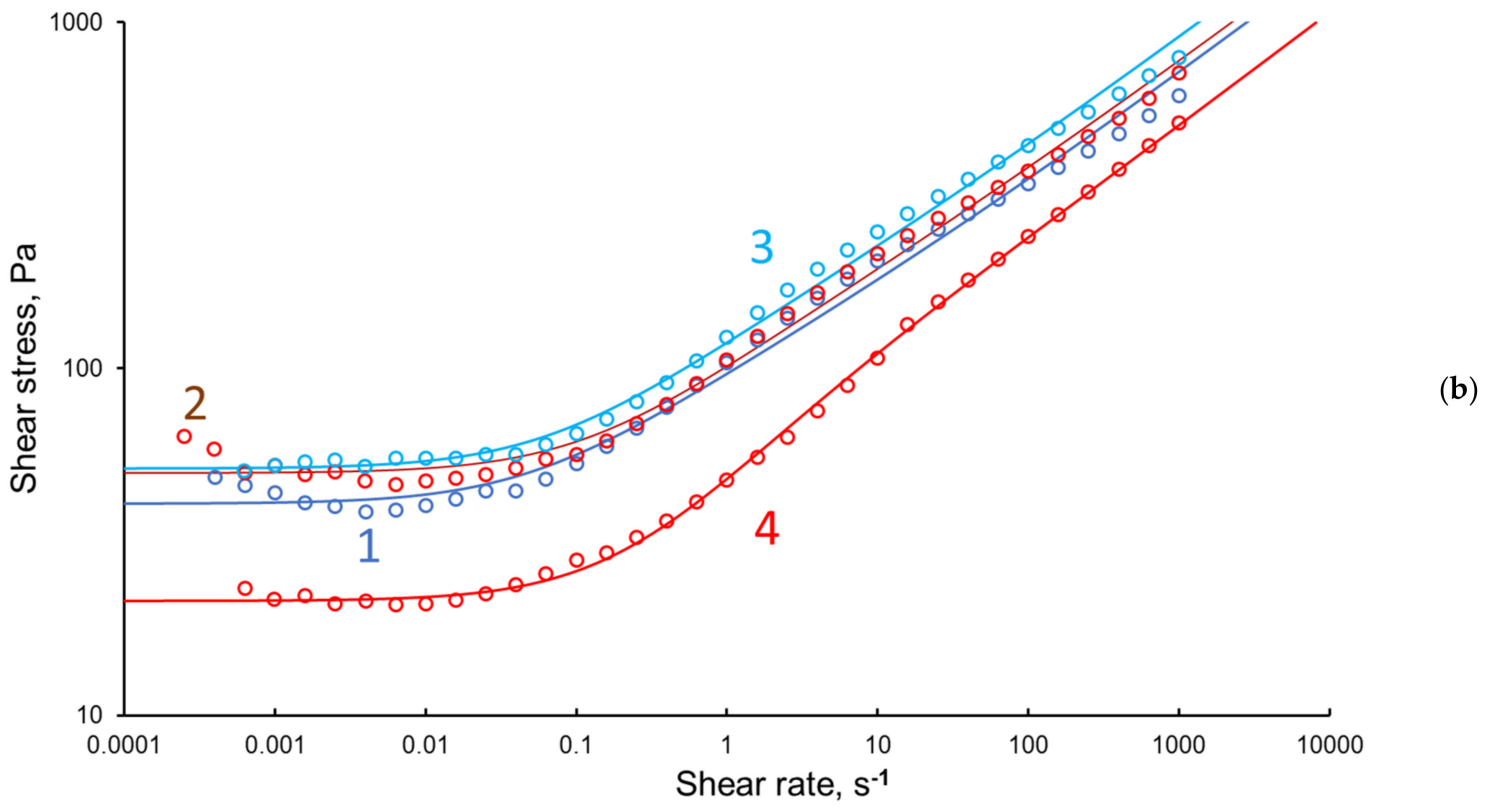
Dependence of viscosity (**a**) and shear stress (**b**) on the shear rate in the shear test (Down SR mode): CRG-HG (1), CRG-CNW-HG-3 (2), CRG-CNW-HG-5 (3), CRG-CNW-HG-10 (4).

The rheological tests of the obtained hydrogels were performed by shear testing with decreasing shear rate (Down SR mode) from 1000 s^−1^ to the lowest possible value (0.0001 s^−1^). The dependence of viscosity on shear rate (Figure 4, Table 2) showed that all samples tested were non-Newtonian fluids with a high-strength supramolecular structure typical of gels.

The Cox–Merz rule (dynamic viscosity is equal to shear viscosity when the values of angular frequency and shear rate are equal) did not hold for the tested samples, so we used only shear test data to analyze the rheological behavior. There is hysteresis when testing with decreasing and increasing strain rates, but it is not significant and only over a narrow range of strain rates, so it can be ignored. The graphs show only shear mode data with a decrease in shear rate.

The contribution of the yield strength is insignificant at high speeds and manifests itself at a low shear rate, but determines the state of the gel. The calculation was performed according to Equation (3) by varying the parameters with an accuracy of 1%; the minimum standard deviation (SD) of the viscosity was the calculation criterion. The lowest Newtonian viscosity is usually the solvent viscosity, but in this case, we take a value of zero (for the viscosities of these samples this does not affect the result, since this value is significant only at high strain rates). The rheological properties of the samples tested are summarized in Table 2. Sample CRG-CNW-HG-5 is characterized by the strongest hydrogel structure (Table 2).

**Figure 5:**
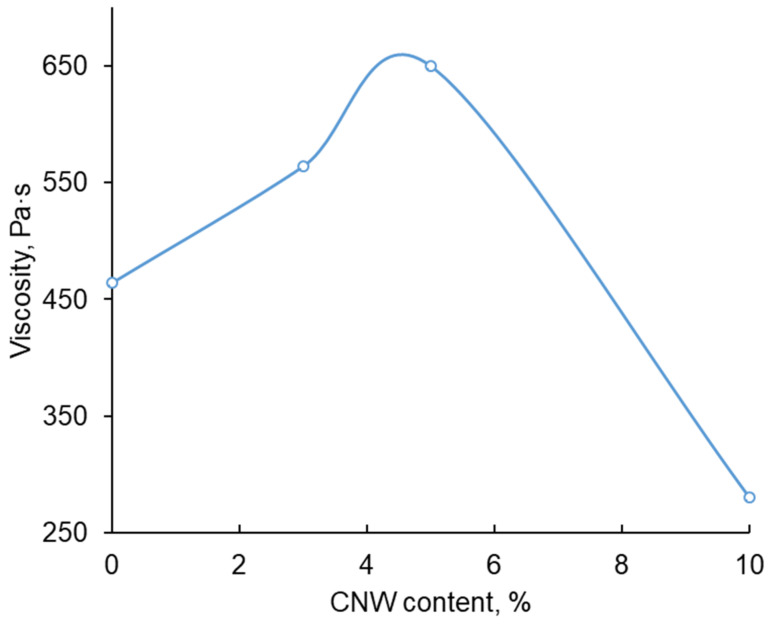
Viscosity as a function of CNW introduced at low shear rates (0.1 s^−1^).

It should be noted that the most pronounced relationships characterizing the strength of the structure of the obtained hydrogels were observed at low shear rates (Figure 5).

The analysis of the obtained results allows us to conclude that the strongest gel is formed at the introduction of 5% CNWs. Increasing the CNW content up to 10% leads to an increase in the number of local crosslinks and a violation of the gel homogeneity.

At the same time, the effect of CNWs on the structural organization of hydrogels depends significantly on the type of functional group and the structural organization of the polyacid. For example, for composite hydrogels based on sodium alginate (which contains weaker carboxyl groups, pKa ~3) and CNWs, we previously found that the introduction of CNWs up to 10% strengthened the hydrogel structure while maintaining its homogeneity [13]. The presence of stronger acidic groups (-OSO_3_H, pKa ~2) in the CRG leads to a different type of interaction with CNWs, and when they are increased to 10%, the homogeneity of the crosslinked structure is broken, and a discrete composite hydrogel is formed.

### 3.4. Properties of CRG-CG and CRG-CNW-CGs

The resulting hydrogels were freeze dried to form cryogel systems: CRG-CG, CRG-CNW-CG-3, CRG-CNW-CG-5, and CRG-CNW-CG-10.

The analysis of the specific surface area of the formed cryogels (Table 3) shows the effect of the amount of CNWs introduced on the structure of the cryogel. When 5% CNWs were introduced, the specific surface area increased insignificantly, and increasing the amount of CNWs to 10% resulted in a significant increase in the specific surface area of the cryogel. This indicates the formation of a new cryogel structure (discrete gel) and these data are in good agreement with the previously described rheological properties of hydrogels (see Section 3.3).

**Figure 6:**
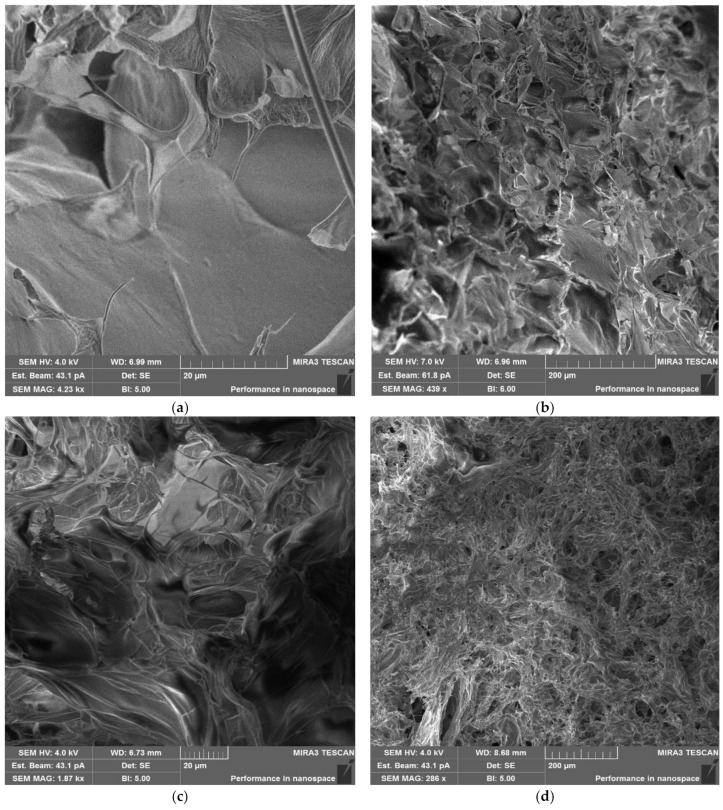

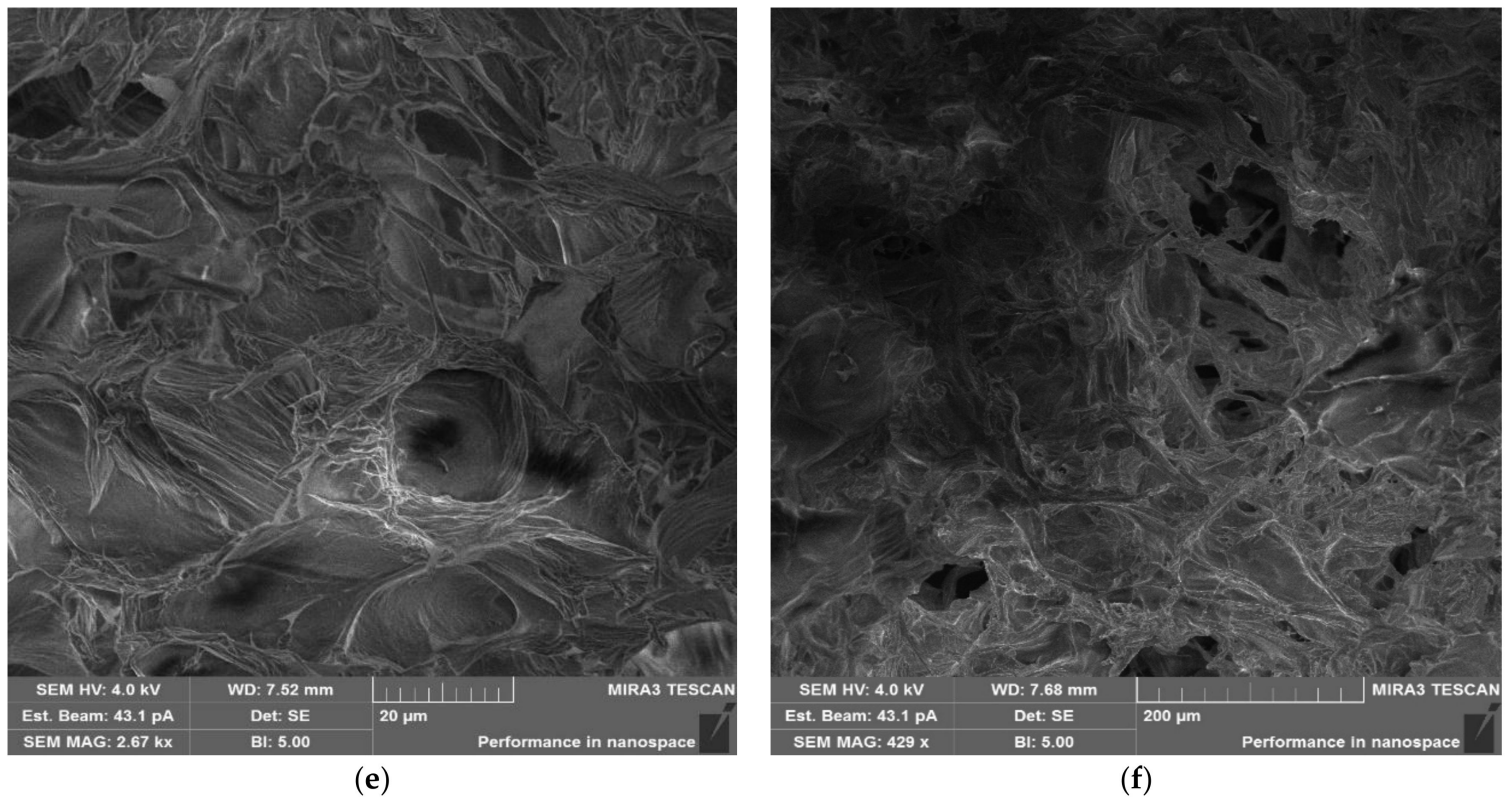
SEM images of cryogels at higher and lower magnification: CRG-CG (**a**,**b**), CRG-CNW-CG-5 (**c**,**d**), CRG-CNW-CG-10 (**e**,**f**).

The analysis of the BET specific surface area of the cryogels was performed using Kr because of the poor escape of N_2_ from the samples. SEM analysis of the cryogel surface morphology revealed a change in the structural organization of the CRG cryogel upon introduction of CNWs. The interaction of positively charged CNWs and negatively charged CRG chains resulted in the formation of more porous structures (Figure 6).

### 3.5. Release of MET from MET-CRG-CG and MET-CRG-CNW-CGs

To test the developed cryogels as potential vaginal delivery systems with modified release, we chose two different systems CRG-CNW-CG-5 (strong structured gel) and CRG-CNW-CG-10 (discrete gel), and loaded them with MET. As a result, the following samples were obtained: MET-CRG-CG, MET-CRG-CNW-CG-5, MET-CRG-CNW-CG-10. The content of MET in the resulting samples was calculated according to the Equation (1) and presented in Table 4.

To properly study drug release from vaginal delivery systems, the physiological conditions of the vaginal environment should be considered. The vaginal fluid contains several antimicrobial substances, including lysozyme, lactoferrin, fibronectin, and polyamines, as well as carbohydrates from epithelial glycogen, amino acids, aliphatic acids, and proteins [49]. In addition, the vaginal microflora consists mainly of Lactobacilli, and the pH of the healthy vagina is acidic (approximately 3.5–4.5) [40,50]. Owen and Katz [51] developed a vaginal fluid simulator (VFS) that models the properties of vaginal secretions, primarily pH and osmolarity, which primarily determine drug release and distribution.

**Figure 7:**
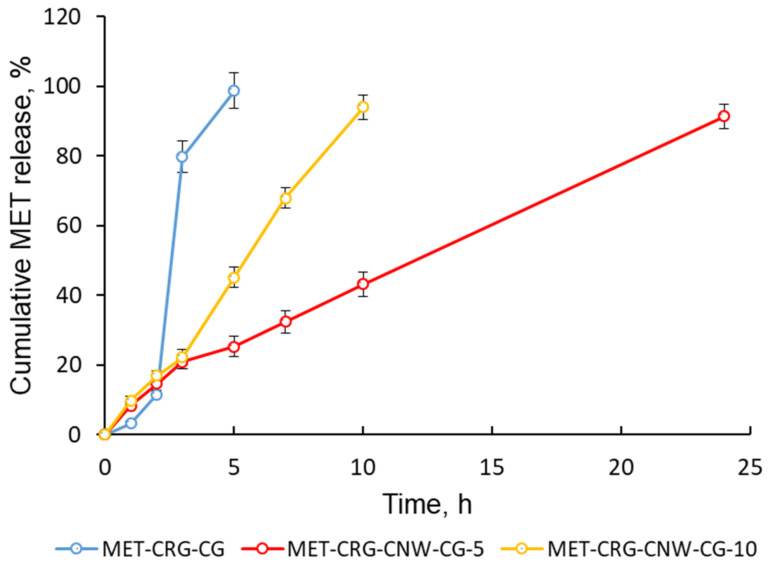
MET release kinetics at 37 °C from the MET-CRG-CG and MET-CRG-CNW-CG in VFS (pH 4.2). Data are presented as mean ± SD (*n* = 3).

The study of MET release kinetics in vitro has shown that the rate and pattern of release depend on the amount of CNWs introduced, which determines the structure of the hydrogel/cryogel (Figure 7). In contrast to MET-CRG-CG, which showed a high rate of MET release within 5 h, cryogels containing 5% CNWs showed a sustained MET release within 24 h. However, the MET release profile of MET-CRG-CNW-CG-10 had a different nature. The amount of MET released was almost directly proportional to time, reaching a maximum in 10 h. This pattern of MET release is related to the structure of the obtained polymer systems. The introduction of 5% CNWs led to the formation of a composite hydrogel/cryogel reinforced with CNWs, but increasing the amount of CNWs to 10% contributed to the formation of a more dispersed (discrete) hydrogel/cryogel and correspondingly increased the rate of drug release. The different nature of drug release from cryogels with different CNW content is in excellent agreement with the rheological properties of the hydrogels and the morphology data of the resulting cryogels.

To evaluate the mechanism of MET release from the prepared polymeric carriers, the cumulative release curves were linearized according to the following mathematical models: zero-order kinetics (Equation (4)), first-order kinetics (Equation (5)), Higuchi (Equation (6)), Korsmeyer–Peppas (Equation (7)), and Peppas–Sahlin (Equation (8)) [45,46,47,48]. The fitting parameters are shown in Table 5.

The kinetics of MET release from cryogels were best described by the Korsmeyer–Peppas and Peppas–Sahlin models (Table 5). According to the Korsmeyer–Peppas model, the release of MET from MET-CRG-CG did not follow Fick’s law (Supercase II transport, *n* > 1). The introduction of 5% CNWs into the CRG resulted in a cryogel with a drug release mechanism following Case II transport/zero-order kinetics (*n* = 1). In this case, the prolongation of MET release was due to swelling of the polymer matrix/relaxation of the macrochains. A further increase in CNW content of up to 10% and destruction of the structured gel matrix increased the effect of relaxation on drug release (*n* = 1.1). The results are consistent with the Peppas–Sahlin model, which showed that polymer swelling and relaxation are the most important factors determining the pattern of MET release (*K*_r_ > *K*_d_).

Thereby, the process of MET release from the developed systems can be represented as follows: diffusion of the solvent into the matrix, swelling, and formation of a gel layer in which the drug is dissolved and then released.

### 3.6. Pharmacological Activity of MET-CRG-CNW-5

Based on the studied release kinetics and structural data, MET-CRG-CNW-CG-5 was selected for testing pharmacological activity against the trichomoniasis pathogen (*T. vaginalis*).

**Figure 8:**
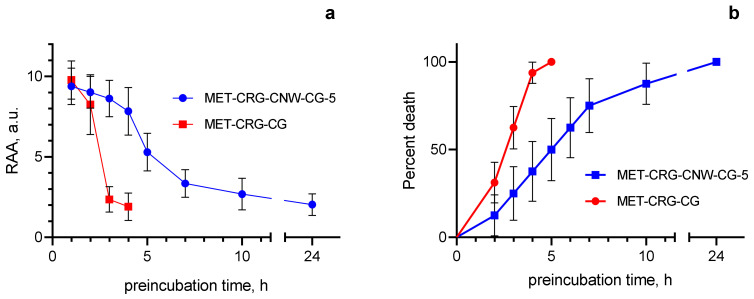
Residual antiprotozoal activity of MET-CRG-CG and MET-CRG-CNW-5 against *T. vaginalis* ATCC 50143 after preincubation of cryogels in TYM (**a**), and comparison of survival curves of *T. vaginalis* ATCC 50143 (**b**). Log-rank (Mantel–Cox) test was used to compare survival curves: χ^2^ = 8.09; df = 1; *p* = 0.0045. Gehan–Breslow–Wilcoxon test: χ^2^ = 5.485; df = 1; *p* = 0.0192. Median survival: MET-CRG-CNW-CG-5—5.5 h; MET-CRG-CG—3 h. Data are presented as mean ± SD (*n* = 9).

MET-CRG-CG was used as a control to evaluate the pharmacological activity of MET-CRG-CNW-5 against *T. vaginalis* ATCC 50143. Overall, the results of this phase of the study were consistent with the data described above (Section 3.6), showing that MET-CRG-CG was significantly different from MET-CRG-CNW-5 in terms of MET release rate; we found that MET-CRG-CNW-5 in TYM had high residual antiprotozoal activity against *T. vaginalis* ATCC 50143 within 24 h (Figure 8a).

For example, after 4 h of pre-incubation in TYM, the residual antiprotozoal activity of MET-CRG-CNW-5 was determined to be 83.4% of the initial level, which in absolute terms was 7.8 ± 1.5 a.u. RAA against *T. vaginalis* ATCC 50143; over the same period, the residual antiprotozoal activity of MET-CRG-CG against this strain decreased to 19.5% of the initial level and was only 1.9 ± 0.9 a.u. RAA. After 5 h of pre-incubation in TYM, MET-CRG-CG completely lost its antiprotozoal activity; at the same time, MET-CRG-CNW-5 retained more than half of the residual antiprotozoal activity (56.4%; 5.3 ± 1.2 a.u. RAA). As a result, after 24 h of pre-incubation of MET-CRG-CNW-5 in TYM, the level of residual antiprotozoal activity was 21.7%, i.e., 2.0 ± 0.7 a.u. RAA against MET-resistant *T. vaginalis* ATCC 50143; this fact indicates the high pharmacological potential of this cryogel.

In addition, differences in the pharmacological activity of MET-CRG-CG and MET-CRG-CNW-5 were confirmed by analyzing the survival curves of *T. vaginalis* ATCC 50143 treated with cryogels preincubated in TYM (Figure 8b). That is, in contrast to MET-CRG-CG, which showed a high rate of MET release and, accordingly, a rapid loss of antiprotozoal activity within 5 h, MET-CRG-CNW-5, which had a sustained release of MET over 24 h, maintained a high level of residual antiprotozoal activity throughout the experiment.

**Figure 9:**
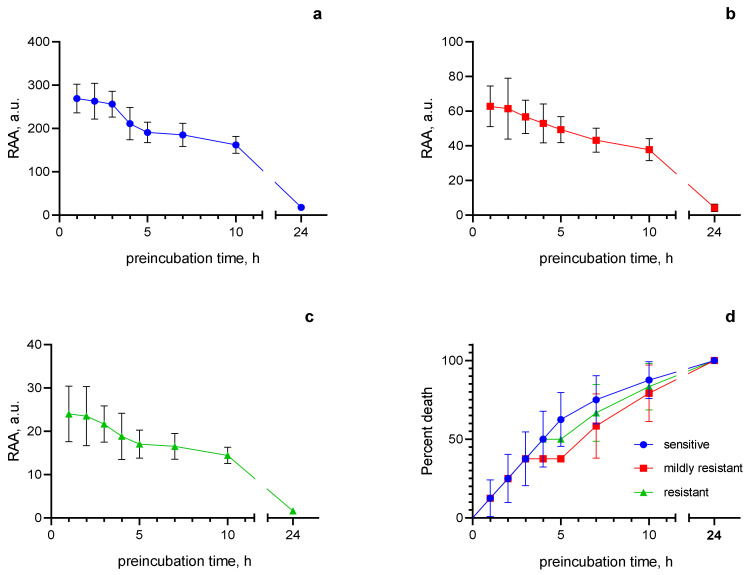
Residual antiprotozoal activity of MET-CRG-CNW-5 against sensitive (**a**), mildly resistant (**b**), MET-resistant (**c**) clinical isolates of *T. vaginalis*, and comparison of *Trichomonas* survival curves (**d**). For comparison of survival curves Log-rank (Mantel–Cox) test: χ^2^ = 0.4002; df = 2; *p* = 0.8187. Log-rank test for trend: χ^2^ = 0,1; df = 1; *p* = 0.7518. Gehan–Breslow–Wilcoxon test: χ^2^ = 0.2476; df = 2; *p* = 0.8836.

To investigate the pharmacological activity of MET-CRG-CNW-5 against clinical isolates of *T. vaginalis*, the sensitivity of these strains to MET was preliminarily determined. According to the sensitivity to MET, all clinical isolates were divided into three groups: sensitive (MIC 0.8 ± 0.2 µg/mL; *n* = 10), mildly resistant (MIC 3.6 ± 0.9 µg/mL; *n* = 15); resistant (MIC 9 ± 2 µg/mL, *n* = 4). Despite differences in the susceptibility of clinical *Trichomonas* isolates to MET, the residual antiprotozoal activity of MET-CRG-CNW-5 remained at a level sufficient to kill all clinical isolates within 24 h (Figure 9a–c).

In addition, no significant differences were found in the pharmacological activity of MET-CRG-CNW-5 against *Trichomonas* with different resistance to MET, as confirmed by the analysis of the survival curves of *T. vaginalis* strains treated with cryogel pre-incubated in TYM (Figure 9d). That is, MET-CRG-CNW-5 has a long-term high level of antiprotozoal activity against all clinical isolates of T. vaginalis used in this study, including those resistant to MET; this fact indicates the high pharmacological efficacy and possible high potential of MET-CRG-CNW-5 for clinical use.

Thus, a long-term preservation (within 24 h) of the antiprotozoal activity of MET-CRG-CNW-5 against clinical isolates of *T. vaginalis* with different levels of susceptibility to MET was found, suggesting the possibility of a wide use of such cryogels as “delivery vehicles” that release the antibiotic over a long period of time and at a constant rate. It seems obvious that such “delivery vehicles” can be effective against pathogens with a wide range of drug resistance, while the possibility of using a combination of different antibiotics in cryogels is not ruled out, but all this requires further research, including in vivo.

## 4. Conclusions

In this work, we prepared a cryogel polymeric carrier based on CRG and CNWs for application as a vaginal delivery system of MET with modified release.

First, we obtained CRG-CNW hydrogels formed by various interactions between CRG macrochains and CNWs: electrostatic interactions in the formation of polyelectrolyte complexes, hydrogen bonding, and entanglement of CRG macromolecules. We showed the effect of CNW concentration on the rheological properties of the formed hydrogels; the strength of CRG hydrogels increased when the CNW content was increased to 5% (in this case, highly structured gel systems were formed) and then dramatically decreased when 10% CNWs were added (due to strong interactions of protonated amino groups of CNWs with sulfate groups of CRG, the system collapsed with the formation of discrete gels).

Second, we prepared cryogels based on the developed hydrogel compositions and tested their properties as a function of CNW content. We found that the CNW-containing cryogels were more porous structures in contrast to the CNW-free system, and the study of the specific surface area confirmed that increasing the CNW content led to the disintegration of the cryogel structure with the formation of a discrete polymer system.

Third, we loaded the developed formulations with the antiprotozoal drug MET and investigated the profile and mechanism of MET release. At the target vaginal pH of 4.2, sustained release of MET occurred in 24 and 10 h for 5% and 10% formulations, respectively. We demonstrated that the nature of the cryogel structure, mediated by the CNW content, influenced the rate and type of drug release. The release curves correlated well with the Korsmeyer–Peppas and Peppas–Sahlin models. The prolonged-release mechanism is mediated by polymer swelling and chain relaxation in the polymer matrix, which makes it possible to program the MET release rate by varying the rheological properties of the hydrogels (viscosity and relaxation time) by changing the CNW content.

Finally, we evaluated the pharmacological activity of the developed cryogels against *T. vaginalis*. We found that MET-CRG-CNW-5 has a long-term (24 h) antiprotozoal effect against *Trichomonas*, including those resistant to MET. These results indicate that cryogels such as MET-CRG-CNW can be used as a basis for the development of new agents for the treatment of infections caused by pathogens with varying degrees of drug resistance.

We suggest that MET-CRG-CNW cryogels could be considered as promising dosage forms for the treatment of vaginal infections.

## Figures and Tables

**Table 1:** Conditions for preparation of CRG-CNW-HGs.

Sample	Mass (g)				
	CRG	Water	0.5% CNW Dispersion	2% Acetic Acid Solution	MET
CRG-HG	0.8	25.0	-	0.2	-
CRG-CNW-HG-3	0.8	20.2	4.8	0.2	-
CRG-CNW-HG-5	0.8	17.0	8.0	0.2	-
CRG-CNW-HG-10	0.8	9.0	16.0	0.2	-
CRG-MET-HG	0.8	25.0	-	0.2	0.267
CRG-CNW-MET-HG-5	0.8	17.0	8.0	0.2	0.267
CRG-CNW-MET-HG-10	0.8	9.0	16.0	0.2	0.267

**Table 2:** Rheological characteristics * of CRG-HG and CRG-CNW-HGs.

Sample	*τ*_0_ (Pa)	*η*_0_ (Pa∙s)	*θ* (s)	*η*_0_/*θ* (Pa)	Relative SD (%)
CRG-HG	40.8	310	9.70	32	9%
CRG-CNW-HG-3	45.0	248	6.25	40	10%
CRG-CNW-HG-5	51.5	308	6.75	46	8%
CRG-CNW-HG-10	21.4	59	1.33	40	3%

* rheological parameters obtained by varying with an accuracy of 1% by the minimum value of the relative standard deviation from the experimental values of the shear test (Down SR mode) at 20 °C ($\eta_{\infty }$ = 0 Pa s, *p* = 0.667 by default).

**Table 3:** Specific surface area of cryogels using the Brunauer, Emmett, and Teller (BET) approach.

Sample	Specific Surface Area (m^2^/g)
CRG-CG	0.624
CRG-CNW-CG-5	0.643
CRG-CNW-CG-10	0.715

**Table 4:** MET content in MET-CRG-CG and MET-CRG-CNW-CG.

Sample	MET Content (μg/mg)
MET-CRG-CG	250
MET-CRG-CNW-CG-5	241
MET-CRG-CNW-CG-10	233

**Table 5:** Fitting parameters of the MET release kinetic models.

Kinetics Model *	Parameter	MET-CRG-CG	MET-CRG-CNW-CG-5	MET-CRG-CNW-CG-10
Zero order Q = *K*_0_ *t* (4)	*r* ^2^	0.8448	0.9636	0.9942
	*K*_0_	19.4	4.0	9.3
First order lnQ = *K*_1_*t* (5)	*r* ^2^	0.6908	0.7887	0.7746
	*K*_1_	53.4	21.9	32.8
Higuchi Q = *K*_H_*t*^0.5^ (6)	*r* ^2^	0.4347	0.9673	0.8179
	*K*_H_	24.4	12.2	19.3
Korsmeyer–Peppas Q = *K*_KP_*t^n^* (7)	*r* ^2^	0.9999	0.9999	0.9973
	*n*	1.86	1.00	1.15
	*K*_KP_	3.2	1.0	7.2
Peppas–Sahlin Q = *K*_d_*t^m^* + *K*_r_*t*^2*m*^ (8)	*r* ^2^	0.9999	0.9948	0.9973
	*m*	0.93	0.34	0.57
	*K*_d_	0.0	0.0	0.0
	*K*_r_	3.2	8.8	7.2

* Q is the MET cumulative release (%); *K*_0_ is the zero-order release constant; *K*_1_ is the first-order release constant; *K*_H_ is the Higuchi constant; *K*_KP_ is the release rate constant; *n*—release exponent, the value of *n* is used to characterize the release mechanism of drug: Fickian diffusion (Case I transport) and diffusion-controlled release (*n* ≤ 0.5); abnormal transport/mixed release mechanism: diffusion + relaxation (0.5 < *n* < 1); Case II transport/zero-order release (*n* = 1) and Supercase II transport (*n* > 1), the release does not obey Fick’s law [52]; *K*_d_ and *K*_r_ are kinetic constants; the values of *K*_d_ indicate the contribution of diffusion (Fickian or Case I kinetics), and the value of *K*_r_ is associated with the dissolution, as well as relaxation of the polymer chains; this model accounts for the coupled effects of Fickian diffusion and Case II transport [53]; *m* is the diffusional exponent; *t* is the time.

## Data Availability

Data available upon request.
